# Antimicrobial photodynamic therapy effects mediated by methylene blue in surfactant medium as an adjuvant treatment of teeth with apical periodontitis and presence of fistula–Protocol for randomized, controlled, double-blind clinical trial

**DOI:** 10.1371/journal.pone.0315169

**Published:** 2024-12-19

**Authors:** Carolina Wince, Claudio T. Kassa, Joaquin Insper, Daniela Amzallag, Wilhem Consonlandich, Ana Carolina A. C. Tortamano, Fabiana Divino Magalhães, Valentina Lestido, Christiane Pavani, Renato A. Prates

**Affiliations:** 1 Universidad Catolica del Uruguay, Montevideo, Uruguay; 2 Postgraduate Program in Biophotonics Medicine Applied to Health Sciences, Universidade Nove de Julho, Sao Paulo, Brazil; University of Insubria, ITALY

## Abstract

**Objective:**

This study aims to evaluate the efficacy of photodynamic therapy as an adjunct to conventional endodontic treatment in patients with apical periodontitis and fistulas. In this study, a fistula is characterized as a pathological conduit originating from the infected region at the root apex of the tooth, traversing the oral mucosa, and extending to the external surface of the gingiva. This pathological condition frequently complicates the management of endodontic infections, thereby necessitating the evaluation of supplementary therapeutic interventions. The standard treatment for endodontic infections involves thorough disinfection of the root canal system to remove microbial contamination from the canal and surrounding tissues. To potentially augment the efficacy of conventional treatment, aPDT is proposed as a supplementary, non-invasive technique. This innovative technique uses a photosensitizer, which is a light-sensitive dye, in combination with a light source to produce reactive oxygen species. Reactive oxygen species can effectively target and eliminate bacteria in the root canal system, potentially enhancing treatment outcomes.

**Methods:**

The study will involve 140 teeth with apical periodontitis and fistulas. The teeth will be randomly assigned to one of two treatment groups. Group I will receive only the conventional endodontic treatment, which includes root canal cleaning, shaping, and obturation. Group II will undergo the same conventional endodontic treatment, but with an additional step of aPDT. The aPDT procedure involves applying a photosensitizer to the root canal and irradiating it with light to produce reactive oxygen species. Each group will consist of 70 teeth to ensure adequate statistical power. The primary outcome is fistula resolution, assessed clinically at 15 and 30 days post-treatment. The secondary outcome is the comparison of apical radiolucency from periapical radiographs to evaluate healing and reduction of periapical pathology.

**Conclusions:**

The study aims to determine if adding aPDT significantly improves the management of apical periodontitis and overall success rates of endodontic treatment. The results will provide insights into the effectiveness of aPDT as an adjunctive treatment and its potential benefits in clinical practice.

## Introduction

Endodontics is a dental specialty concerned with the etiology, prevention, diagnosis, and management of pathological conditions affecting the dental pulp and their consequences in the periapical region. One of the fundamental principles is the prevention of infection and its control when present. When dental pathology is established, involving the periapical tissues and causing inflammation, it is classified as apical periodontitis. At this stage, the inflammatory response triggers the degradation of periapical tissues due to pathogens associated with the pulp infection. A fistula generally arises from an injury, frequently due to an infection. A dental fistula is a narrow tract that forms from the infected region of the tooth, typically at the root apex, to the external surface of the oral mucosa. This tract acts as a conduit for microorganisms and their byproducts. As the tract becomes filled with exudate, a localized edema may occur on the mucosal surface [1].

Antimicrobial photodynamic therapy—aPDT is a non-invasive treatment modality and ca be used as an adjunct to conventional periodontitis treatment. The objective of the therapy is to eliminate microbial cells through the significant production of reactive oxygen species. This technique targets both bacteria and fungi. The therapy operates through the interaction of a specific light wavelength with a photosensitizer (PS) in the presence of oxygen [2, 3].

The mechanism of action can be described by the Jablonski diagram. The photosensitizer (PS) absorbs visible light, promoting it from the ground singlet state to the singlet excited state, which has a brief lifetime. In this state, the PS can lose energy through non-radiative processes such as fluorescence or undergo intersystem crossing to the triplet state, which has a longer lifetime. From the triplet state, the PS may lose energy via phosphorescence or participate in the generation of reactive oxygen species (ROS) through two distinct mechanisms: Type I reaction, which involves electron transfer to produce free radicals, or Type II reaction, where energy is transferred to molecular oxygen to form singlet oxygen. The occurrence of Type I and Type II reactions is influenced by various factors, including the physicochemical properties of the photosensitizer (PS), such as its quantum yield and absorption spectrum, as well as the characteristics of the microenvironment, including the availability of molecular oxygen and the presence of solvents or other reactive species. [4].

Therefore, aPDT is utilized as an adjunctive treatment in endodontics. This non-invasive approach involves the application of a photosensitizer, which, when activated by a specific wavelength of light, generates reactive oxygen species. These reactive species effectively target and eradicate bacterial cells, including those within biofilms, enhancing the overall effectiveness of endodontic therapy [2, 3, 5, 6].

Phenothiazines are planar amphipathic tricyclic molecules featuring a quaternary intrinsic nitrogen atom and exhibit phototoxic activity against bacteria. In dentistry, phenothiazines are frequently employed as photosensitizers, with methylene blue (MB) and toluidine blue (TB) being key examples. These compounds are heterocyclic dyes comprising two benzene rings conjugated to a nitrogen atom and a sulfur atom. They exhibit significant absorption when exposed to visible light in the 600–660 nm range, which enhances their efficacy in generating reactive oxygen species within biological tissues [4, 7, 8].

The use of methylene blue in antimicrobial photodynamic therapy has demonstrated reliable outcomes. The hydrophilic nature of MB, combined with its low molecular weight and positive charge, facilitates its penetration through the cell walls of microorganisms [9].

It is essential to acknowledge that the antimicrobial efficacy of antimicrobial photodynamic therapy is contingent upon the match between the absorption spectrum of the photosensitizer and the emission spectrum of the light source. The effectiveness is influenced by factors such as the wavelength of the light, irradiance, exposure duration, and the absorption characteristics of the PS [10].

The aggregation of the photosensitizer is another factor influencing the efficacy of aPDT. This phenomenon, known as dimerization, can be controlled using surfactants. Sodium dodecyl sulfate (SDS) has been demonstrated to effectively reduce this aggregation [11]. Some researchers have demonstrated that methylene blue exhibits greater antimicrobial efficacy when dissolved in ethanol or urea solutions compared to water-based solutions. The addition of surfactants, such as SDS, has been shown to mitigate MB aggregation. This results in an increased concentration of monomeric MB and a reduction in aggregate formation, due to enhanced electrostatic and hydrophobic interactions among MB molecules [9, 12].

The use of methylene blue in a SDS solution at a 0.25% resulted in more effective microbial reduction during aPDT against *Candida albicans* compared to MB alone. The study found that MB, when in its monomeric form, was a more potent photosensitizer, with Type II photochemical reactions—involving singlet oxygen—being the most effective mechanism for inactivating *C*. *albicans* [11].

Following the disruption of the biofilm during endodontic therapy, residual bacterial populations may persist within the periodontal pockets, necessitating adjunctive antimicrobial treatment. Successful endodontic treatment requires precise canal instrumentation, effective irrigation, and appropriate intracanal medication. This process encompasses both manual and mechanized techniques, the application of irrigants, intracanal medicaments, and root canal sealers, all of which involve considerable time and resources. Antimicrobial photodynamic therapy (aPDT) may serve as an adjunctive treatment modality for periodontal disease, potentially reducing reliance on systemic antimicrobials and decreasing the risk of developing bacterial resistance [9].

Thus, the aim of this protocol is to evaluate the photodynamic effect of methylene blue in 0.25% SDS for the treatment of apical periodontitis in presence of fistula. The hypothesis to be tested is that both interventions will exhibit equivalent efficacy, with no statistically significant difference. The evaluation will focus on the resolution of the fistula and the improvement of radiographic indicators, assessed at 15 and 30 days post-endodontic treatment for apical periodontitis [13].

## Materials and methods

### Study design

This study will be a two-arm, single-center, double-blind, randomized controlled clinical trial with a 1:1 allocation ratio. Upon approval of the study protocol by the Research Ethics Committee of the Universidad Catolica del Uruguay—UCU, the trial will be conducted involving participants with apical periodontitis and a fistula in single-rooted teeth. Inclusion criteria require initial periapical radiography demonstrating apical radiolucency and fistulography to confirm the presence of the fistula. Recruitment will occur at the University Health Clinic of UCU, where the study will be conducted. Participants will be required to read, understand, and sign the Free and Informed Consent Form, which has been approved by the Research Ethics Committee. (211103b).

All participants will receive treatment from a single experienced clinician, who will oversee data collection and procedural execution. Initial periapical radiographs will be required for all participants. Root canal preparation will be conducted using a rotary endodontic device (DENTSPLY). A diode laser apparatus (DMC, THERAPY EC, Sao Carlos, Brazil) with a wavelength of 660 nm, power output of 100 mW, and an irradiation duration of 3 minutes will be utilized [14].

Participants and outcome evaluators will remain blinded to the assigned interventions throughout the study. Unblinding will be permitted only under exceptional circumstances where knowledge of the actual treatment is critical for the ongoing management of the participants.

All participants will receive conventional treatment as described below. Additionally, the study group will undergo antimicrobial photodynamic therapy (aPDT), while the control group will receive a simulation of the laser application. During this process, both participants and the operator will be required to wear protective eyewear.

### Interventions

All participants will be treated by the same experienced endodontist, who will oversee both data collection and procedural management. Each participant will receive conventional treatment.

A baseline periapical radiograph will be required for all participants, as well as follow-up radiographs at 15- and 30-days post-treatment. These radiographs will be obtained by a different operator, who will analyze them. This operator will be blinded to the intervention received by each participant.

Conventional treatment will be performed using the Protaper rotary system (Dentsply Maillefer rotary instruments). The canal irrigation will be with 5% sodium hypochlorite, 17% ethylenediaminetetracetic acid, and 2% chlorhexidine [14]. Subsequently, a methylene blue solution in a surfactant medium will be applied using a size 30 paper point cone (Dentsply), with a pre-irradiation time of 1 minute. The study group will receive a 3-minute irradiation period, while the control group will be administered the photosensitizer along with a simulated laser application. This simulation will be achieved by placing an aluminum foil barrier at the tip of the laser equipment to block the light. During these procedures, both the operator and participants will wear protective eyewear to ensure eye safety and concealment. Calcium hydroxide will be applied to the root canals as a long-term intracanal dressing for endodontic treatment.

A diode laser device (Therapy EC, DMC, São Carlos, Brazil) with a wavelength of 660 nm and a power output of 100 mW will be used, according to the parameters detailed in Table 1. The pre-irradiation and irradiation durations will be identical for both groups.

**Table 1:** Dosimetric parameters.

PARAMETERS	
Wavelength (nm)	660
Operating mode	Continuos
Power (mW)	100
Irradiance (mW/cm^2^)	250
Target irradiated area (cm^2^)	0.4
Exposure time (s)	180
Radiant exposure (J/cm^2^)	10
Energy (J) per point	18
Number of irradiated points	2
Application technique	Entry duct and fistula
Number of sessions	1

The photosensitizer used will be methylene blue at a concentration of 0.005%, dissolved in 0.25% sodium dodecyl sulfate.

### Inclusion criteria

A single root tooth with apical periodontitis and fistula; periodontal pockets ≤ 4 mm; ≥ 18 years of age and not taking antibiotics.

### Exclusion criteria

Cancer, diabetes, coagulation diseases, anemia; orthodontic treatment; pregnant women or lactating women and the impossibility to use the rubber dam isolation technique.

### Ethics and dissemination

The study will be conducted in accordance with Decree 158/19 of the Executive Branch, which regulates research involving human subjects in Uruguay. Additionally, the study will comply with Law 18,331 (Law on the Protection of Personal Data and the Action of "Habeas Data").

The Research Ethics Committee of Universidad Católica del Uruguay approved the protocol (211103b). Additionally, the protocol was registered under number NCT06413836 in the Clinical Trial Protocol Registration and Results System. The results from this trial will be presented at conferences and published upon completion of the study.

**Fig 1:**
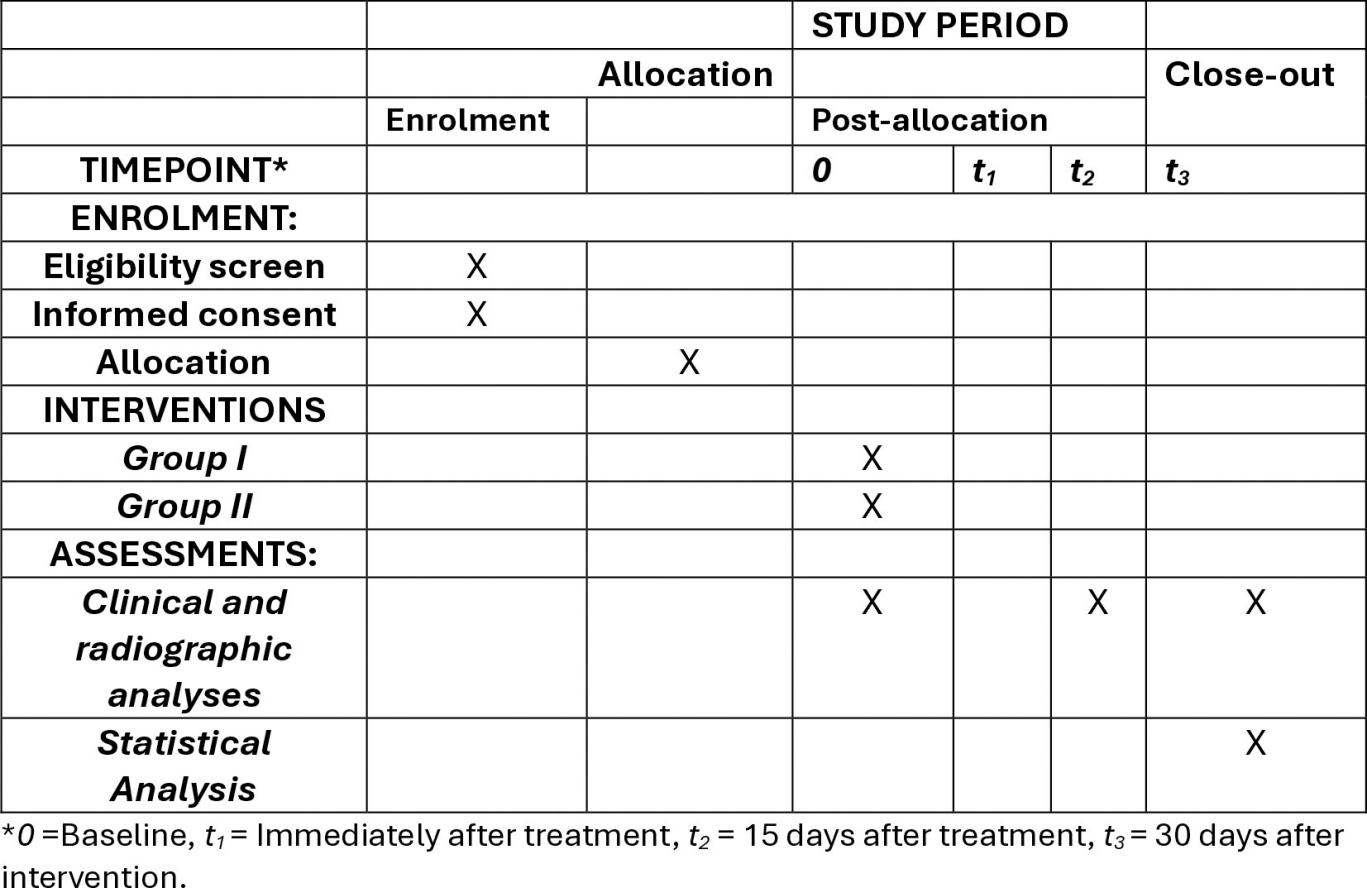
SPIRIT figure recommended by 2013 SPIRIT statement.

The study is anticipated to last up to 20 months, beginning from the recruitment date. The protocol was developed in accordance with the SPIRIT guidelines (Standard Protocol Items: Recommendations for Interventional Trials) (Fig 1), and the SPIRIT checklist is provided as an additional file. Upon completion of the study, the CONSORT guidelines (Consolidated Standards of Reporting Trials) will be adhered to for reporting.

Recruitment will commence following the acceptance of the protocol for publication.

Participants will be informed that they may withdraw from the study at any time and for any reason if they choose to do so. Researchers may also remove participants from the study if deemed necessary.

### Sample size calculation

The sample size calculation was conducted with the statistical software G-Power 3.1 (Universität Kiel, Germany) applying the chi-square homogeneity test. Identical sample sizes were determined for both the aPDT group and the sham group. A type I error rate of 5% and a statistical power of 80% were specified [15], resulting in a required sample size of n = 70, accounting for a 10% loss to follow-up. The sample size calculation was based on established literature, and a significance level of 5% will be employed [8, 13, 16].

### Randomization

Participants will be selected and randomly assigned to one of two experimental groups. Randomization will be carried out by an investigator who is not directly involved in participant treatment, using the website www.randomizer.org. The randomization results will be communicated through opaque, sealed envelopes containing information about whether the treatment is laser-based or conventional. These envelopes will be securely stored and will not be accessible to the observing researcher. Prior to treatment, the operator responsible for administering the therapy will receive the envelope to determine the assigned treatment group.

### Blinding

Participants and outcome evaluators will be blinded to the assigned interventions. Unblinding will be permitted only under exceptional circumstances where knowledge of the actual treatment is crucial for the continued management of the participants.

## Outcome variables

### Primary outcomes

• Resolution of the Fistula: The primary outcome will be the resolution of the fistula, assessed at 15- and 30-days post-treatment. This will be evaluated based on clinical examination and radiographic evidence.

• Radiographic Improvement: The improvement in radiographic signs of apical periodontitis will be assessed at 15- and 30-days post-treatment, focusing on changes in the periapical radiolucency.

### Secondary outcomes

• Comparison of Apical Radiolucency: The secondary outcome will involve comparing the apical radiolucency in periapical radiographs of teeth with healed fistulas. This will be assessed before the intervention and at 15 and 30 days post-treatment, focusing on changes in the radiographic appearance of periapical lesions [13].

### Statistical analysis

The distribution of data within each group will be checked. The categorical variables will be analyzed using the Pearson Chi-square test or two sample t-test. The correlation between clinical or radiographic findings and the healing fistula will be analyzed by Pearson Chi-square test or Mann Whitney U test [13].

## Discussion

aPDT is considered a complementary approach in endodontic treatment. A primary limitation of this technique is the formation of compounds that can reduce the efficacy of the therapy. This group has been studying these compounds for several years [12, 17, 18].

On the other hand, SDS demonstrated the ability to mitigate the effect of dimer formation [19]. Therefore, this study aims to evaluate the effectiveness of methylene blue combined with 0.25% SDS in photodynamic therapy for treating apical periodontitis with fistulas. The objective is to assess how effectively this combination eliminates persistent microorganisms in root canals [8].

This study addresses the challenge of treating teeth with fistulas, which often require several clinical sessions. The main difficulty is effectively eliminating the bacteria that reside within the root canals of these teeth [4].

aPDT is a non-invasive therapeutic modality that can be employed as an adjunctive treatment for apical periodontal diseases. It reduces the reliance on systemic antimicrobials, thereby mitigating the risk of antimicrobial resistance associated with these medications.

Unlike antibiotics, aPDT exhibits low toxicity to humans and is not associated with the development of bacterial resistance [20]. Given the issue of antibiotics and their indiscriminate use by patients, it is essential to seek alternative and adjunctive methods for combating microorganisms. This is particularly pertinent in countries where, despite legislation regulating antibiotic sales, patients can still obtain these medications easily without prior consultation or authorization from a healthcare professional.

## Supporting information

S1 File SPIRIT checklist. (PDF)

S2 File Statement consent in original language. (DOCX)

S3 File Statement consent in English. (DOCX)

S4 File Cosentimiento informado. (DOCX)
