# Supplementary material for: Antimicrobial photodynamic therapy effects mediated by methylene blue in surfactant medium as an adjuvant treatment of teeth with apical periodontitis and presence of fistula–Protocol for randomized, controlled, double-blind clinical trial
Source: PLoS One. 2024 Dec 19;19(12):e0315169. doi: 10.1371/journal.pone.0315169 (PMC11658630; doi:10.1371/journal.pone.0315169)
Supplement: S2 File — (DOCX) [file pone.0315169.s002.docx]

**UNIVERSIDAD CATOLICA DEL URUGUAY**

**UNINOVE – UCU**

**CAROLINA WINCE GONZALEZ**

**STUDY PROTOCOL**

ANTIMICROBIAL PHOTODYNAMIC THERAPY EFFECTS MEDIATED BY METHYLENE BLUE IN SURFACTANT MEDIUM AS AN ADJUVANT TREATMENT OF TEETH WITH APICAL PERIODONTITIS AND PRESENCE OF FISTULA – PROTOCOL FOR RANDOMISED, CONTROLLED, DOUBLE-BLIND TRIAL

**ABSTRACT**

When we talk about Endodontics, we refer to the science and art encompassing the etiology, prevention, diagnosis, and treatment of pathological alterations of the dental pulp and their repercussions on the periapical region and, consequently, on the entire body. One of its key pillars is infection control—preventing and treating it when it occurs.

When dental disease has already developed, affecting the periapical tissues and causing inflammation, we refer to apical periodontitis. At this stage, inflammation begins, accompanied by the destruction of periapical tissue caused by the agents responsible for pulp infection. The presence of a fistula is often the result of a lesion. Infections or inflammations can lead to the formation of a fistula, also known as an intraoral sinus tract. A dental fistula is a small channel that forms from the infected area of the tooth—usually at the root apex—to the outer surface of the gum. This passage acts as a reservoir for microorganisms and their byproducts, and as it fills, a small bump forms on the gums.

When the pathology has developed and needs treatment, endodontic treatment comes into play. This includes manual, mechanized, or combined techniques, alongside the use of irrigants, intracanal medications, and canal sealers. These procedures require time, materials, and are often not entirely satisfactory. For this reason, antimicrobial photodynamic therapy (aPDT) has emerged as an adjunct in endodontic treatment. aPDT is a non-invasive treatment that uses a photosensitizer and a light source to generate reactive oxygen species, which lead to bacterial death. However, the main limitation of this technique is the formation of dimers, which reduces its effectiveness. On the other hand, sodium dodecyl sulfate (SDS) has shown the ability to reduce dimer formation.

The purpose of this study is to validate the photodynamic effect of methylene blue delivered in 0.25% SDS for the treatment of patients with apical periodontitis and fistula, aiming to eradicate persistent microorganisms in root canals and improve the outcomes of such treatments.

The methodology will involve selecting 30 teeth diagnosed with apical periodontitis and fistula. Patients will be randomly assigned to two groups as follows:

- **Group I**: Patients undergoing conventional root canal treatment (n = 15).
- **Group II**: Patients undergoing conventional root canal treatment combined with antimicrobial photodynamic therapy (n = 15).

Clinical findings, such as the absence of symptoms, fistula presence, and radiographic parameters, will be evaluated and recorded.

.

KEYWORDS: periodontitis, fistula, endodontic treatment, antimicrobial, photodynamic therapy, methylene blue.

1. **CONTEXTUALIZATION**

**From the moment we discuss non-vital teeth in which the disease is already established, we consider the microbial factor.** Microbes in the canal lumen are planktonic (1). The biofilm adheres to the canal walls, much like it adheres to the surfaces of teeth. When there is a periradicular lesion, there is often a significant amount of biofilm present.

Evidence highlights the polymicrobial etiology of endodontic infections, where bacteria and their byproducts are the main agents driving the progression and spread of apical periodontitis (2). The microbial factors within the root canals extend to the apical tissues, triggering chronic inflammatory responses. Consequently, apical periodontitis results from a complex interaction between microbial factors and the host's defense mechanisms (3).

When endodontic infection becomes chronic, it may drain to the gingival surface through an intraoral communication known as a sinus tract or fistula (1). This epithelial-lined passage sometimes extends to a superficial opening or stoma on the attached gingiva. Generally, a periapical infection with an associated sinus tract is not painful, although discomfort may occur before the sinus tract develops (4). In addition to providing a channel for releasing infectious exudate, alleviating pain, the presence of the fistula can help identify the origin of a specific infection.

The fistula can be located adjacent to or at a distance from the infection's source. The trajectory of the sinus tract provides objective information for identifying the tooth causing the infection (1,4). To trace this path, a procedure called fistulography is performed, which involves inserting a gutta-percha cone of a calibrated size into the tract's opening until resistance is felt. A radiographic image is then captured, revealing the trajectory of the cone and identifying the tooth causing the infection.

The mechanism of bacterial action is confined to the root canal, not the bone, emphasizing the importance of cleaning the canal. At this point, the importance of cleaning and shaping the canal system becomes evident (5).

The most recommended treatment for eliminating a fistula is endodontics, aiming to drain the affected area and administer appropriate medication beforehand. Endodontics involves performing a deep cleaning of the affected area to eliminate any remaining infected tissue (4,1).

Successful endodontic therapy requires the elimination of microorganisms and biofilm through root canal disinfection methods. Irrigation is defined as washing a cavity with a liquid. The goals of irrigation in endodontics are both mechanical and chemical (5). Sodium hypochlorite (NaOCl), ethylenediaminetetraacetic acid (EDTA), and chlorhexidine (CHX) are the most common effective irrigants for removing microorganisms from the coronal and middle thirds of dental canals, although their efficacy significantly decreases in the apical third (6).

The intracanal medication of choice is chlorhexidine mixed with calcium hydroxide, as studies have demonstrated the enhanced properties of this combination. In recent years, new alternative treatment modalities have been proposed, including high-power lasers and antimicrobial photodynamic therapy (PDT) (7). Photodynamic therapy has been proposed as a new complementary method for additional disinfection of the root canal system, potentially improving treatment outcomes (8). This technique is based on the use of photosensitizing substances that bind to cells and are activated by light of a suitable wavelength, promoting microorganism death.

A photosensitizer is a light-sensitive chemical product (9). Using a photosensitizer for the photodynamic treatment of infected tissues can allow absorption by bacterial cells and irradiation of the tissues, potentially resulting in the destruction of both bacteria and infected tissues (10). For this study, PDT was validated for endodontic treatment using a photosensitizer, methylene blue, at a concentration of 10 mg/ml, and diode laser irradiation at a wavelength of 665 nm and a power of 100 mW (11). The use of methylene blue in photodynamic therapy has demonstrated very promising results.

The hydrophilic nature, low molecular weight, and positive charge of methylene blue enable its passage through microbial walls (12). This process, based on the use of photosensitizers that bind to cells and are activated by light of a suitable wavelength, promotes microorganism death through the formation of reactive oxygen species. Phenothiazine dyes are the most used in dentistry, with methylene blue being the most prevalent.

It is important to emphasize that aPDT’s antimicrobial effect occurs only when the photosensitizer's absorption spectrum and the emitted radiation are compatible. Wavelength, light intensity, exposure time, and the photosensitizer's absorption capacity determine the results (13). Therefore, the treatment's effectiveness depends on optimizing these numerous parameters.

Another significant factor affecting PDT's effectiveness is the photosensitizer's aggregation, which reduces its efficacy at higher concentrations. This effect, known as dimerization, can be managed by using surfactants. Consequently, sodium dodecyl sulfate (SDS) can improve the photochemical conditions, maximizing the therapeutic potential of aPDT (14).

After reviewing numerous previous studies, it is evident that aPDT should not replace conventional endodontic treatment procedures but should be considered as an adjunct. Research has confirmed that low-intensity lasers are safe, easy to handle, widely accepted by patients, and promote antimicrobial activity when combined with a photosensitizing dye.

It is indisputable that proper endodontic treatment, including correct shaping, irrigation, and intracanal medication, is essential for treatment success. However, improving the technique always aims to provide better-quality treatment (15).

This study aims to evaluate the reduction in bacterial load following conventional endodontic treatment with and without antimicrobial photodynamic therapy in teeth with apical periodontitis and fistula.

The study will use diode laser equipment (DMC, THERAPY EC, São Carlos, Brazil) with a wavelength of 660 nm and a power of 100 mW for 3 minutes.

1. **OBJECTIVE**

To evaluate the action of aPDT as an adjuvant in the endodontic treatment of teeth with apical periodontitis and the presence of a fistula.

1. **HYPOTHESIS**

The use of aPDT demonstrates greater antimicrobial effectiveness in the treatment of teeth with apical periodontitis and the presence of a fistula compared to conventional endodontic treatment alone.

1. **METHODOLOGY**

After this research project is submitted for approval by the Ethics Committee for Research at UCU, this clinical trial will be conducted on patients with apical periodontitis and the presence of a fistula in single-rooted teeth. The selected teeth must meet the following criteria: an initial periapical radiograph showing apical radiolucency and fistulography to confirm the presence of a fistula.

Participants will be recruited at the University Health Clinic of the Catholic University of Uruguay, where all clinical procedures for the study will be performed. Participants must read, understand, and sign the informed consent form approved by the UCU Ethics Committee for Research.

The clinical parameters to be analyzed will include the evaluation of periapical radiographs to assess apical radiolucency and the presence of a fistula.

A total of 30 participants will be selected and randomly divided into two experimental groups:

- **Group 1:** Mechanized endodontic treatment with calcium hydroxide (CaOH) as intracanal medication between sessions. This medication is routinely used in all conventional endodontic treatments.
- **Group 2:** Mechanized endodontic treatment with calcium hydroxide (CaOH) as intracanal medication, preceded by the application of antimicrobial photodynamic therapy with methylene blue. Methylene blue is a substance with extensive studies supporting its efficacy in the oral cavity, and its active ingredient is already used in endodontic treatments.

For aPDT, methylene blue will be used as a photosensitizing agent at a concentration of 0.005%. It will be applied inside the canal using a sterile paper cone for 3 minutes, followed by laser light administration for 40 seconds (wavelength: 660 nm, energy density: 4 J/cm², power: 100 mW) in direct contact at the entrance of the root canal.

**4.1 TREATMENT PROTOCOL**

Current periapical radiographs will be required for all participants. All participants will be treated by an experienced endodontist, who will be responsible for data collection and performing the procedures.

For the treatments, a rotary endodontic motor (Dentsply) will be used to perform the preparation of the root canals.

A diode laser equipment (DMC, THERAPY EC, São Carlos, Brazil) with a wavelength of 660 nm and a power of 100 mW will be used for 3 minutes.

**4.2 INCLUSION CRITERIA**

Participants must have a single-rooted tooth with apical periodontitis and the presence of a fistula.
All selected teeth must not show signs of periodontal disease, meaning they should not have periodontal pockets greater than 4 mm in depth.
Participants must be over 18 years old.

**4.3 Exclusion criteria**

Participants under 18 years old.
Participants with comorbidities such as cancer, diabetes, coagulation disorders, anemia.
Participants undergoing orthodontic treatment.
Pregnant women or breastfeeding patients.
Participants with teeth that cannot be subjected to absolute isolation.

**4.4 Endodontic clinical evaluation**

The participants will be pre-examined for the evaluation of endodontic parameters through the use of periapical radiographs with radiographic positioning devices, to determine the presence of apical radiolucency, as well as clinically confirming the presence of a fistula.

**4.5 Antimicrobial Photodynamic Therapy**

After the endodontic treatment, aPDT will be applied using a diode laser equipment (DMC, THERAPY EC, São Carlos, Brazil) with a wavelength of 660 nm and a power of 100 mW for 3 minutes.

**4.6 Statistical analyses**

The distribution of the data within each group and the homogeneity of the variables will be verified to determine whether an analysis of variance (ANOVA) or an alternative test should be used. Once this information is obtained, the most appropriate statistical test will be performed.

The sample size calculation is based on the literature, and a significance level of 5% will be adopted.

**5. Activity schedule**

|  | 1°sem/2021 | 2°sem /2021 | 1°sem/ 2022 | 2°sem/ 2022 | 1°sem/ 2023 | 2°sem/ 2023 |
| --- | --- | --- | --- | --- | --- | --- |
| Literature review | X | X | X | X | X | X |
| Submission to the Ethics Committee |  | X |  |  |  |  |
| Participants selection |  | X | X |  |  |  |
| Clinical procedures |  | X | X | X | X |  |
| Data collection |  | X | X | X | X |  |
| Statistical analysis |  |  | X | X | X |  |
| Writing and submission of the article |  | X | X | X | X | X |
| Defense |  |  |  |  |  | X |

**Bibliographic References**

1. Cohens Pathways of the pulp, Kenneth M Hargreves, Louis H, Berman , undécima edición Cap 14 y 15 2016
2. Singh S, et al Photodynamic therapy: An adjuct to convetional root canal desinfection strategies. Endodontic Journal 2015
3. Aguinaldo Silva Garcez et al. Antimicrobial effects of photodynamic therapy on patients with necrotic pulps an periapical lesión. Journal Endodontics 2008 feb
4. Baumagartner JC, Picket AB, Muller JT. Microscopoc examination of oral sinus tratcts and their associated periapical lesions. Journal Endodontics 1984; 10:146
5. Basrani B, Ghanem A, Tjaderhane L. Physical and chemical properties of clorhexidine and calcium hydroxide- containing medicatios. Journal Endodontics 2004;30:413
6. Asnaasharim et al Comparision of Antibacterial Effects of Photodynamic Therapy and an Irrigation Avtivation System on Root Canals infected with enterococus faecalis: an in vitro study J Lasers Med Sci 2020
7. Soukos NS, et al Photodynamic Therapy for endodontic desinfection, Jpurnal ENdodontic 2006
8. Plotino G et al. Photodynamic therapy in enodontics. Int Endodontics Journal 2019
9. Tortamano ACAC, Anselmo GG, Kassa CT, Godoy-Miranda B, Pavani C, Kato IT, et al. Antimicrobial photodynamic therapy mediated by methylene blue in surfactant vehicle on periodontopathogens. Photodiagnosis Photodyn Ther. May2020
10. Er Karaoglu G, Ugugr Ydin Z, Erdonmez D, Gol C, Durmus M. Efficacy of antimicrobial photodynamic therapy administred usinf methylene blue, tolouide blue and tetra 2-mercaptoprydine substituted zinc phthalocyanine in root canal contaminated with Enterococcusaecalis. Photodiagnosis Photodyn Ther, december 2020
11. Mozayeni MA, Vatandoost F, Asnaashari M, Shokri M, Azari-Marhabi S, Asnaashari N. Comparing the efficacy of toloudine blue, methylene blue and curcum in photodynamic therapy against enterooccus faecalis. Journal Lasers Med Sci 2020
12. Alvarenga LH, Prates RA, Yoshimura TM, Kato IT, Suzuki LC, Ribeiro MS,et al. Aggregatibacter actinomyceremcomitans biofilm can be inactivated by methylne blue mediated photdynamic therapy. Photodiagnosis Photdyn Ther, march 2015; 12 (1): 132-6
13. Firmino RT, et al Endodontic treatment associates with photodynamic therapy: Case report Photodyn Ther 2016
14. Asnaashari M, Homayuni H, Pymanpour P. The antibacterial effect of additional photodynamic therapy in failed endodontically treated teeth: a pilot study. Journal Lasers MEd Sci. 2016; 7(4) 238-42
15. Anagnostaki E,et al. Systematic review on the Role of Lasers in Endodontic Therapy: Valuable Adjunct Treatment. Dental Journal 2020
