# Supplementary material for: Antimicrobial photodynamic therapy effects mediated by methylene blue in surfactant medium as an adjuvant treatment of teeth with apical periodontitis and presence of fistula–Protocol for randomized, controlled, double-blind clinical trial
Source: PLoS One. 2024 Dec 19;19(12):e0315169. doi: 10.1371/journal.pone.0315169 (PMC11658630; doi:10.1371/journal.pone.0315169)
Supplement: S3 File — (DOCX) [file pone.0315169.s003.docx]

STATEMENT CONSENT

“I…………………………………………………………CI………………………….., have been informed that the researchers Carolina Wince Gonzalez CI 3303978-9 (Professor at the Catholic University of Uruguay) and Renato Prates (Professor at the Universidade Nove de Julho de São Paulo, Brazil). They want to carry out a research entitled EVALUATION OF THE EFFECT OF THERAPY PHOTODYNAMICS MEDIATED BY METHYLENE BLUE CARRIED IN THE MEDIUM SURFACTANT AS A ADJUVANT IN THE TREATMENT OF PARTS WITHAPICAL PERIODONTITIS AND PRESENCE OF FISTULA RANDOMIZED CLINICAL TRIALS, DOUBLE BLIND CONTROLLED. As part of the information that the researchers have provided I have understood that:

1- This research will take place at the University Health Clinic, of the University

Catholic of Uruguay and in the private office of Dr. Carolina Wince Gonzalez. The objective of this research is to evaluate the effectiveness of photodynamic therapy used as an adjuvant in the endodontic treatment of teeth with periodontitis apical and presence of fistula.

2- I have been informed that I will not have any discomfort or discomfort other tan

conventional treatment, as well as economic differences with it.

3- I understand that being part of any of the 2 groups of which you will be a part

I will randomly receive conventional endodontic treatment, and if I am part of the

Laser treatment will only increase the treatment time by 5 min.

4- I am also informed that I will not suffer any physical, emotional or economical as it is part of the research project, since the apparatus used. It is fully approved and is used in dental procedures in a manner safe.

5- If I have any questions before, during or after the treatment, I will contact you

with Dr. Carolina Wince Gonzalez, principal investigator of the project, on your pone 099637248 or email caritowince@gmail.com.

6- My participation in the research project is completely voluntary and I can

leave at any time without any harm, my treatment will be completed anyway.

7- I was informed that my data will remain anonymously, as well as data obtained from the research.

8- I was informed that I will be able to access the results of the investigation once I have the project is completed and they are published.

9- As well as they informed me that this research has been approved by the UCU Research Ethics Committee whose official website address Committee where I can corroborate this point is: http://www.ucu.edu.uy/comite_etica

10- Having understood the information detailed above, and being able to carry out all the questions that arise regarding the procedures before and during the treatment, I agree to participate in these research.

________________________ ____________________________

Signature of the participant Signature of the responsible researcherI

C-I C.I

Date Date
