# Supplementary material for: Antimicrobial photodynamic therapy effects mediated by methylene blue in surfactant medium as an adjuvant treatment of teeth with apical periodontitis and presence of fistula–Protocol for randomized, controlled, double-blind clinical trial
Source: PLoS One. 2024 Dec 19;19(12):e0315169. doi: 10.1371/journal.pone.0315169 (PMC11658630; doi:10.1371/journal.pone.0315169)
Supplement: S4 File — (DOCX) [file pone.0315169.s004.docx]

COSENTIMIENTO INFORMADO

“Yo………………………………………………………CI………………………….., he sido informado que los investigadores Carolina Wince Gonzalez CI 3303978-9 (Profesora de la Universidad Católica del Uruguay ) y Renato Prates (Profesor de la Universidade Nove de Julho de San Pablo, Brasil) quieren llevar a cabo una investigación titulada EVALUACION DEL EFECTO DE LA TERAPIA FOTODINAMICA MEDIADO POR AZUL DE METILENO VEHICULIZADO EN MEDIO SURFACTANTE COMO COADYUVANTE EN EL TRATAMIENTO DE PIEZAS CON PERIODONTITIS APICAL Y PRESENCIA DE FISTULAS ENSAYO CLINICO RANDOMIZADO, CONTROLADO A DOBLE CIEGO. Como parte de las informaciones que los investigadores me han proporcionado yo he entendido que:

1. Esta investigación tendrá lugar en la Clínica Universitaria de la Salud, de la Universidad Católica del Uruguay y en el consultorio particular de la Dra. Carolina Wince Gonzalez. El objetivo de esta investigación es evaluar la efectividad de la terapia fotodinámica usada como coadyuvante en el tratamiento endodóntico de piezas con periodontitis apical y presencia de fistula.
2. Se me ha informado que no tendré incomodidades ni molestias diferentes al tratamiento convencional, así como tampoco diferencias económicas con el mismo.
3. Entiendo que siendo parte de cualquiera de los 2 grupos de los cuales se formara parte al azar recibiré el tratamiento endodóntico convencional, y si formo parte del tratamiento con laser solo se aumentara el tiempo de tratamiento en 5 min.
4. Así también se me informo que no tendré ningún perjuicio físico, emocional ni económico al ser parte del proyecto de investigación, ya que la aparatología utilizada esta totalmente aprobada y es utilizada en procedimientos odontológicos de forma segura.
5. Ante cualquier duda antes durante o después del tratamiento me pondré en contacto con la Dra. Carolina Wince Gonzalez investigadora principal del proyecto a su teléfono 099637248 o a su email caritowince@gmail.com
6. Mi participación del proyecto de investigación es totalmente voluntaria y puedo abandonar en cualquier momento sin tener perjuicio ninguno, mi tratamiento será culminado de todas maneras.
7. Se me informo que mis datos permanecerán en el anonimato, así como también los datos que se obtengan de la investigación
8. Fui informado que podre acceder a los resultados de la investigación una vez haya culminado el proyecto y los mismos sean publicados
9. Así como también me informaron que esta investigación ha sido aprobada por el Comité de Ética en Investigación de la UCU cuya dirección del sitio web oficial del Comité dónde podre corroborar dicho extremo es: <http://www.ucu.edu.uy/comite_etica>
10. Habiendo entendido las informaciones antes detalladas, y pudiendo realizar todas las interrogantes que me surjan al respecto de los procedimientos antes y durante el tratamiento, acepto participar de la investigación titulada: EVALUACION DEL EFECTO DE LA TERAPIA FOTODINAMICA MEDIADO POR AZUL DE METILENO VEHICULIZADO EN MEDIO SURFACTANTE COMO COADYUVANTE EN EL TRATAMIENTO DE PIEZAS CON PERIODONTITIS APICAL Y PRESENCIA DE FISTULAS ENSAYO CLINICO RANDOMIZADO, CONTROLADO A DOBLE CIEGO.

Firma del participante Firma del investigador responsable

CI CI

Fecha Fecha
